# Validity of constructed‐response situational judgement tests in health professions education: A systematic review and meta‐analysis

**DOI:** 10.1111/medu.70245

**Published:** 2026-06-04

**Authors:** Alexander MacIntosh, Colin Henning, Safea Altef, Ivy Erickson, Sobia Shariff Hussaini, Elizabeth Morgan, Yoon Soo Park, Amir H. Sam

**Affiliations:** ^1^ Research Acuity Insights Inc. Toronto Ontario Canada; ^2^ Applied Modelling and Quantitative Methods Trent University Peterborough Ontario Canada; ^3^ Department of Psychology, Emotion and Health Research Laboratory Trent University Peterborough Ontario Canada; ^4^ Department of Physician Assistant Studies University of Wisconsin–La Crosse La Crosse Wisconsin USA; ^5^ Admissions and Academic Practice Partnerships, Physician Assistant Program Wake Forest University School of Medicine Winston‐Salem North Carolina USA; ^6^ School of Medicine Imperial College London London UK; ^7^ Department of Medical Education, College of Medicine University of Illinois Chicago Illinois USA

## Abstract

**Introduction:**

Constructed‐response situational judgement tests (CR‐SJTs) are used internationally to assess personal and professional attributes in health professions admissions, with over one million applicants to more than 500 programs having used them in the last decade. Despite this, a synthesis of their validity is lacking. This study aimed to quantify the association between CR‐SJT scores and measures of personal, interpersonal and professional performance in health professions education and to determine how moderating factors influence this relationship.

**Methods:**

MEDLINE, EMBASE, CINAHL, ERIC, SCOPUS, Web of Science and grey literature sources (ProQuest Dissertations & Theses, EThOS and OpenGrey) were searched from inception to 7 April 2025. Search was supplemented by contacting experts and admissions directors to include unpublished quality assurance studies. Eligible studies evaluated a CR‐SJT for applicants or trainees in health professions programmes and reported a quantitative relationship with a non‐academic outcome (e.g., professionalism and communication). Of 463 full‐texts reviewed, 27 met inclusion criteria. Following the Preferred Reporting Items for Systematic Reviews and Meta‐Analyses (PRISMA) guidelines, two reviewers independently extracted data and assessed risk of bias using the Quality In Prognosis Studies (QUIPS) tool. A multilevel random‐effects meta‐analysis was used to pool Fisher z‐transformed correlation coefficients. Meta‐regressions tested moderator effects, and sensitivity analyses examined bias impact. The primary outcome was the correlation between CR‐SJT scores and measures of interpersonal or professional skills. Moderators included the construct congruence between the CR‐SJT and the outcome measure, outcome type, publication type, and outcome assessment stage.

**Results:**

The 27 studies yielded 100 unique effect sizes and were judged, in total, to be at moderate risk of bias. The pooled correlation between CR‐SJT scores and outcomes was *z* = 0.22 (95% CI, 0.16–0.28; *p* < 0.001). Construct congruence was the only significant moderator; more congruent outcomes showed *z* = 0.32 (95% CI, 0.25–0.37), compared to that of less congruent outcomes (*z* = 0.17; 95% CI, 0.12–0.18). Publication bias was insignificant (Egger's test, *p* = 0.73).

**Discussion:**

The use of CR‐SJTs in health professions selection is supported by the evidence. Validity depends substantially on construct congruence between the CR‐SJT and the outcome measure. Programs should consider CR‐SJTs within their operational context and with deliberate attention to downstream evaluation alignment.

## Abstract 2

Constructed‐response situational judgment tests (CR‐SJTs) are increasingly used in admissions. How valid are they? Our new meta‐analysis synthesizes the evidence base. #MedEd

## 1 INTRODUCTION

Admissions committees across the health professions have increasingly recognized the need to evaluate personal and professional attributes alongside academic metrics during selection. These attributes are regarded as essential for effective clinical practice and successful progression through training. Strong personal and professional competencies have been associated with improved patient outcomes and greater job satisfaction among healthcare professionals.^1^, ^2^ Consequently, many health professions education programs have placed greater emphasis on assessing these attributes at selection.^3^ However, methods used to assess these attributes at selection vary widely in their reliability, validity and operational feasibility.

Historically, personal and professional attributes have been assessed through tools like personal statements and reference letters. While widely used, these tools have demonstrated poor utility. Specifically, a review from Patterson et al.^4^ identified references and personal statements as being among the least effective tools in selection due to their limited reliability and validity. These limitations predate the current era of generative artificial intelligence (GenAI), wherein students can readily adopt large language models (LLMs) to draft submission materials. Admissions officers and educators are increasingly unable to reliably distinguish between student and GenAI‐authored reflections.^5^, ^6^ As a result, assessments that require real‐time, structured reasoning and judgement may offer a more realistic evaluation of personal and professional attributes, especially in proctored environments.

One method increasingly used to assess personal and professional attributes in selection is the situational judgement test (SJT). SJTs are standardized, low‐fidelity simulations that assess applied judgement across multiple scenarios. This approach reflects the theoretical premise that situational performance is a stronger predictor of future behaviour than retrospective self‐description.^7^ By evaluating responses to standardized situations rather than self‐reported intentions or curated narratives, SJTs operationalize judgement in context, which may also reduce susceptibility to social desirability and impression management.^8^ SJTs have shown acceptable reliability and incremental validity over academic metrics and other selection tools,^4^, ^9^ further supporting their use as a more psychometrically robust and scalable method for assessing personal and professional attributes. However, SJTs represent an assessment methodology rather than a single instrument. Their content, response format, scoring approach and targeted competencies can vary substantially. Kepes et al.^8^ identify SJT design as a major determinant of psychometric quality and highlight the need to document their effects on outcomes. SJT construction and importantly their construct congruence^10^ with anticipated outcomes play a vital role in evaluating validity.

SJTs used in health professions education traditionally employ a fixed‐response format. In these assessments, examinees are presented with a scenario and asked to rate, rank, or select responses from a set of predetermined options.^8^ Meta‐analytic evidence from Webster et al indicates that SJTs demonstrate validity for ‘one‐off assessments’ and ‘longer‐term clinical training or work performance’.^9^ The authors report a pooled correlation of *r* = 0.32 (95% CI 0.26–0.39) across 50 validity coefficients drawn from 26 studies, with individual correlations ranging from 0.04 to 0.72. This evidence largely reflects fixed‐response implementations, with only one of the 50 validity coefficients derived from a constructed‐response SJT (CR‐SJT). Because SJT scores depend partly on test design, validity evidence from one format may not generalise to others, particularly when response processes, scoring methods and construct representation differ.

Since Webster et al., the adoption of CR‐SJTs in health professions admissions has grown rapidly. For instance, the technical manual for the Casper CR‐SJT reports that the assessment has been completed by over one million applicants since 2015 and has been used by more than 500 academic programs internationally.^11^ In contrast to fixed‐response SJTs, CR‐SJTs require examinees to generate their own responses to situational prompts (through written or video‐recorded answers) rather than selecting among predefined options. These responses are typically rated using structured scoring guides applied by trained evaluators.^12^ This shift from response recognition to response generation may alter the psychological processes involved, shifting the reliance on underlying components such as knowledge, cognitive ability or personality.^8^ Prior assessment research suggests that constructed‐response formats may enhance applicant perception,^13^ perceived authenticity^14^, ^15^ and allow for broader representation of candidate competencies,^12^ though this evidence has been derived across various assessment contexts.

At the same time, constructed‐response formats introduce additional measurement challenges that may offset these potential advantages. Constructed‐responses may be influenced not only by construct‐relevant reasoning or articulation, but also by factors like writing quality and fluency, introducing construct‐irrelevant variance.^16^ Evaluators may attend to both substantive content (e.g., engagement with the dilemma and perspective‐taking) and surface features of responses, such as phrasing and flow.^12^, ^17^ These nuances can introduce variability in how answers are interpreted and evaluated against scoring guidelines.

Together, these differences in response generation and scoring processes suggest that the psychometric properties observed for fixed‐response SJTs may not generalize to constructed‐response implementations. Despite this growing prevalence in high‐stakes selection, there has not yet been a dedicated systematic review and meta‐analysis examining the validity of the CR‐SJT format for health professions education.

### 1.1 Objective

This study aims to provide the first meta‐analytic evidence specific to CR‐SJTs for health professions education programmes. Specifically, we quantify the association between CR‐SJT scores and downstream measures of personal, interpersonal and professional performance in educational and clinical contexts. While the ideal validation of any selection tool includes long‐term professional practice outcomes, the current landscape focuses on outcomes within the selection and training phases. This orientation reflects the operational validity and predictive utility prioritised by admissions practitioners. We also examine whether this association varies based on the degree of construct congruence between the CR‐SJT and the outcome measure, the type of outcome and participant characteristics such as demographic background where available.

## 2 METHODS

### 2.1 Study design, reporting guideline and protocol registration

This systematic review and meta‐analysis were conducted in accordance with the Preferred Reporting Items for Systematic Reviews and Meta‐Analyses (PRISMA) 2020 reporting guideline. The protocol was published^18^ and prospectively registered in PROSPERO (CRD42022314561).

### 2.2 Research team and reflexivity

The research team represents a deliberate partnership between industry scientists and independent academic/clinical admissions leaders from Canada, the United States, and the UK. We recognize that our diverse roles influenced the study's conceptualization. To ensure an objective evaluation of CR‐SJTs, we intentionally utilized a ‘critical friends’ model,^19^ acknowledging that one author (AM) is a scientist in industry. Most co‐authors are not active users of a CR‐SJT, and others (AHS, YSP) have an established history of critical inquiry into admissions metrics, fostering a culture where industry perspectives were openly and regularly challenged.

While this research utilises secondary analysis of published data, the inclusion of three active Admissions Directors shifted our focus from purely statistical effect sizes toward operational validity. This practitioner lens ensured that results were interpreted through the practicalities of admissions workflows and the systemic impact of policy changes. Furthermore, our varied geographic and institutional backgrounds shaped our interpretive lens, recognising that validity statistics do not exist in isolation and must be considered within the context of a complex education selection and developmental process.

### 2.3 Data sources and search strategy

A comprehensive search of the published and unpublished literature was conducted in MEDLINE (via OVID, 1948 onward), EMBASE (via OVID, 1980 onward), CINAHL, ERIC, SCOPUS, and Web of Science. Grey‐literature sources included OpenGrey, ProQuest Dissertations & Theses, and EThOS. To minimise publication bias, we additionally contacted subject‐matter experts and admissions directors to identify unpublished reports (e.g., white papers, technical quality assurance studies, conference presentations and papers). Reference lists of included articles and relevant systematic reviews were screened for additional eligible studies. Searches were last updated on 7 April 2025. Detailed electronic search strategies for each database appear in Supporting Information S1‐Detailed Search Strategies.

### 2.4 Eligibility criteria

Eligible studies included applicants to, or trainees in, health professions programmes including but not limited to medicine, nursing, dentistry, pharmacy, physical or occupational therapy and veterinary medicine. Studies were required to evaluate CR‐SJTs, defined as free‐text or video‐recorded response formats designed to assess personal, interpersonal or professional characteristics. Eligible outcomes included quantitative measures of aligned attributes, such as professionalism, communication, teamwork, leadership and workplace‐based performance evaluations. We included primary empirical studies employing observational, experimental, programme evaluation or unpublished quality assurance designs.

We excluded studies of participants outside health professions programmes, those focused solely on faculty or continuing professional development and those that investigated only fixed‐response SJTs or tools measuring exclusively cognitive or academic skills (e.g., science GPA and total MCAT). Outcomes that assessed academic knowledge, procedural or technical skills or constructs unrelated to professionalism and interpersonal attributes were excluded. Reviews, commentaries, editorials, protocols and qualitative studies that did not evaluate the CR‐SJT performance against an outcome were also excluded.

### 2.5 Study selection

After removing duplicates, all titles and abstracts were screened, followed by full‐text review of potentially eligible records by two reviewers independently using Covidence.^20^ Disagreements were resolved through discussion or adjudication by a third reviewer. Reasons for exclusion at full text were recorded. The full process is illustrated in the PRISMA flow diagram (Figure 1).

**FIGURE 1:**
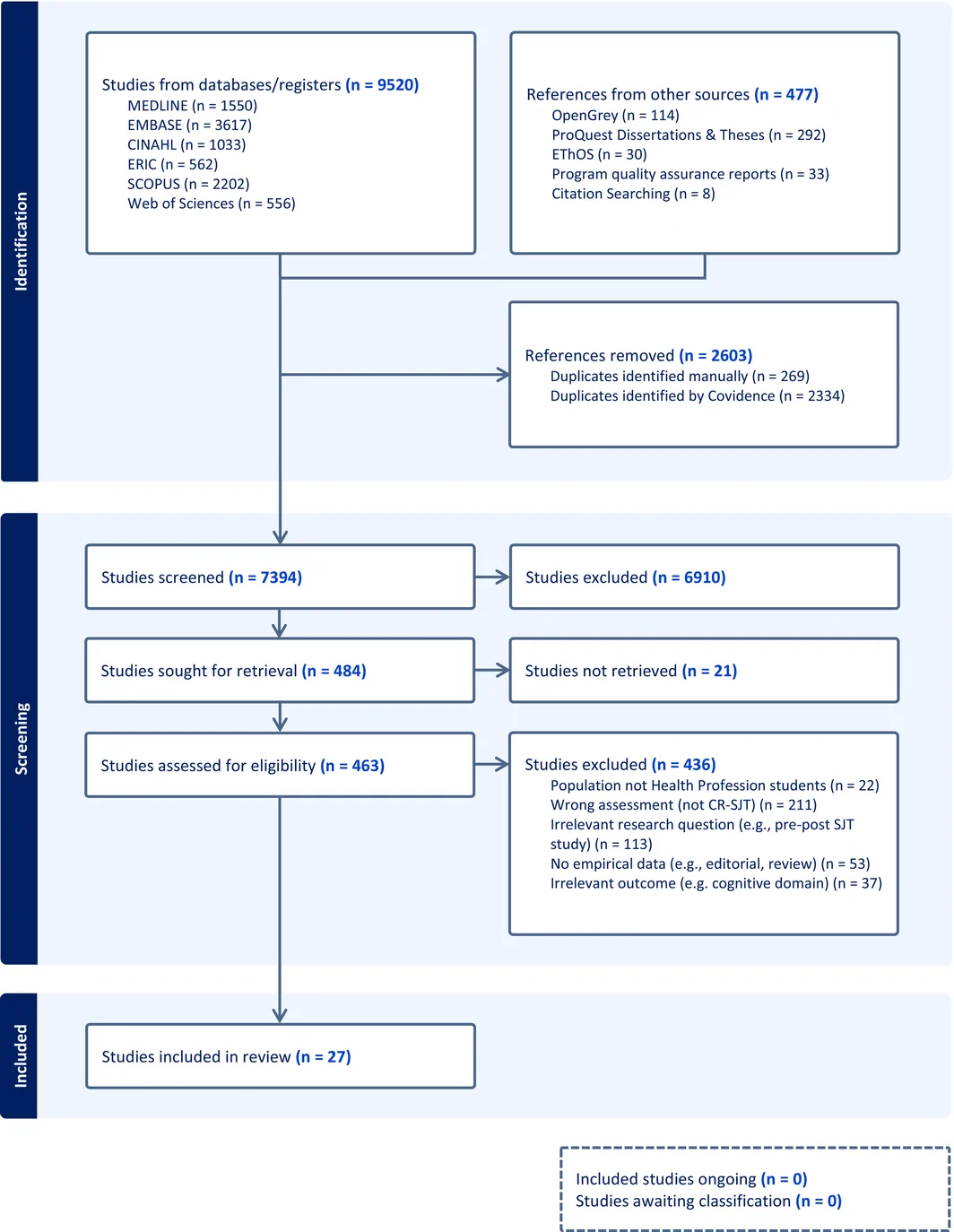
PRISMA flow diagram. PRISMA flow diagram showing identification, screening, eligibility assessment, and inclusion of studies for the systematic review and meta‐analysis of constructed‐response situational judgement tests (CR‐SJTs) in health professions education. Database searches yielded *n* = 7394 records after deduplication. After title and abstract screening, *n* = 463 full‐text articles were assessed for eligibility, resulting in 27 studies (100 effect sizes) included in the final quantitative synthesis. Reasons for exclusion at each stage are shown (e.g., incomplete reporting, overlapping samples, ineligible test format). [Color figure can be viewed at wileyonlinelibrary.com]

A standardized extraction form was piloted and refined. Two reviewers independently extracted study‐level data including setting, participant demographics, sample size, CR‐SJT format and scoring method, outcome type and effect‐size data (see Supporting Information eTable 2‐Characteristics of Included Studies and Supporting Information eAppendix 1‐Citations of Included Studies). Extracted values were verified by cross‐checking and consensus.

### 2.6 Risk of bias assessment

Risk‐of‐bias (RoB) was independently assessed by two reviewers using the Quality in Prognosis Studies (QUIPS) framework across six domains (study participation, attrition, prognostic factor measurement, outcome measurement, confounding and statistical analysis/reporting). Discrepancies were resolved by consensus. Each of the six QUIPS domains was rated as low, moderate or high RoB based on the guidance and example provided by Hayden et al.^21^ Following the approach used by Webster et al, overall study‐level rating was assigned as follows: low if < 2 domains were rated moderate or higher, moderate RoB if 2 or 3 domains were rated moderate or higher, and high RoB if 4 or more domains were rated moderate or higher.^9^ Overall RoB are presented in Supporting Information eTable 3‐QUIPS Risk‐of‐Bias Assessments for Included Studies.

### 2.7 Effect size definition

Effect size coefficients between SJT scores and measures of personal, interpersonal, and professional attributes were extracted from each study. All reported estimates (e.g., Pearson's r, Spearman's ρ, odds ratios, Cohen's d, standardized β) were Fisher z–transformed prior to analysis,^22^ where unique data for each outcome assessment and participant cohort (year) was available.

### 2.8 Statistical analysis

As multiple effect sizes from individual studies were included in the meta‐analysis, a multilevel random‐effects meta‐analysis was conducted using restricted maximum likelihood estimation, with effect sizes at level 1, within‐study variance modelled at level 2 and between‐study variance modelled at level 3.^23^ The pooled effect size was calculated with 95% CIs and *p*‐values. Statistical heterogeneity was tested using the *I* ^2^ statistic and examining the width of 80% credibility intervals. Sensitivity analyses were performed to explore the impact of different sample sizes and RoB by removing studies that were judged to have small sample sizes (*N* < 100) or at high RoB. Publication bias was assessed through multiple methods as recommended by Kepes et al.,^24^ including visual inspection of a funnel plot, the trim and fill approach, the regression‐based Egger's test and testing publication type as a moderator in a meta‐regression. Analyses were conducted using JASP.^25^

To examine heterogeneity in effect sizes, univariate and multivariate meta‐regressions were conducted using prespecified moderators:

- outcome construct congruence (i.e., degree of overlap between the constructs assessed by the CR‐SJT and those captured by the outcome measure; higher congruence was assigned when multiple or most of the outcome score was derived from interpersonal or professional competencies assessed by the CR‐SJT, whereas lower congruence was assigned when one or few components of the outcome score overlapped with CR‐SJT constructs, as is typical of outcomes with a predominantly academic or technical focus; full coding criteria and decisions are provided in Supporting Information eAppendix 2‐Codebook for Outcome Construct Congruence and Supporting Information eTable 4‐Log for Outcome Construct Congruence),
- outcome type (selection evaluations, curricular assessments, workplace evaluations, behavioural outcomes, personality instruments; see Supporting Information eAppendix 3‐Outcome Type Descriptions and Examples),
- publication type (peer‐reviewed, grey literature, quality assurance report), and
- outcome assessment stage (applicant or trainee)

No demographic moderators identified in the protocol were possible to analyse due to a lack of reporting in individual studies.

## 3 RESULTS

### 3.1 Study selection

The search yielded 7394 records, of which 463 underwent full‐text review. Twenty‐seven studies met inclusion criteria, contributing 100 unique effect sizes (Figure 1). Of these, 10 were peer‐reviewed journal articles, 11 were grey literature sources (conference abstracts, theses) and 6 were internal quality assurance reports. Sample sizes ranged from 23 to 7076. Outcome types included selection evaluations (*n* = 41), curriculum and examination performance (*n* = 28), workplace evaluations (*n* = 17), behavioural or professionalism measures (*n* = 7) and personality instruments (*n* = 7). Study‐level details are provided in Supporting Information eTable 2‐Characteristics of Included Studies.

### 3.2 Meta‐analysis effect and subgroup and meta‐regression analyses

Across the 100 effect sizes, the random‐effects multilevel meta‐analysis model estimated a pooled correlation of *z* = 0.22 (95% CI, 0.16–0.28; *p* < 0.001). Substantial heterogeneity was present (*I* ^2^ = 96%).

Subgroup analyses demonstrated that predictor‐outcome construct congruence significantly moderated the validity coefficients. For more congruent outcomes, the pooled correlation was 0.32 (*n* = 22, 95% CI, 0.23–0.40; *p* < 0.001). For less congruent outcomes, the pooled correlation was 0.17 (*n* = 78, 95% CI, 0.11–0.24; *p* < 0.001). Forest plots of individual study effect sizes for each group are presented in Figure 2a,b, and Figure 3 compares the pooled correlations. In contrast, neither publication type, outcome type nor outcome assessment stage significantly predicted effect size across both univariate and multivariate meta‐regressions. Meta‐regression results are detailed in Table 1.

**FIGURE 2:**
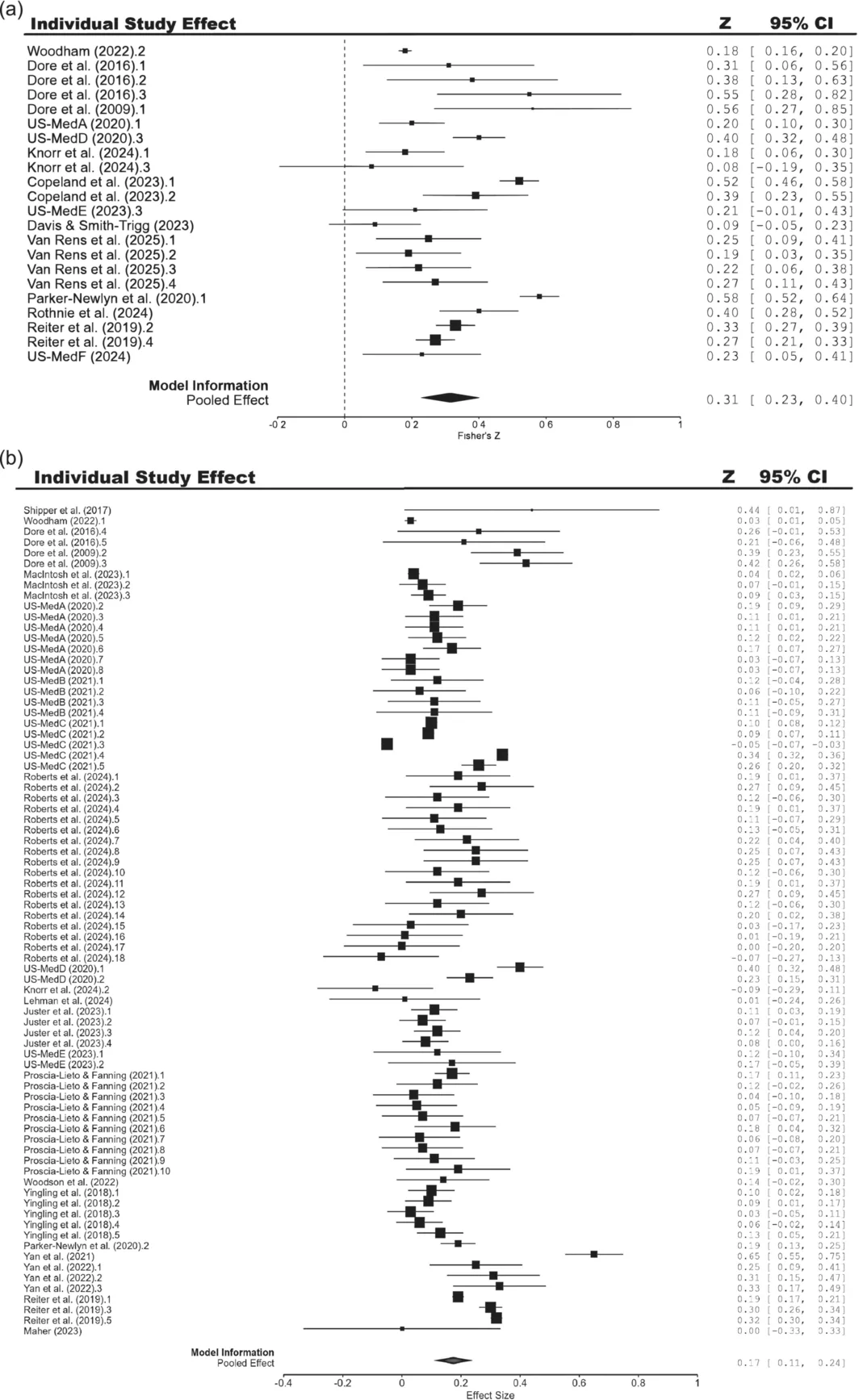
(a) Forest plot of effect sizes for outcomes with higher construct congruence. Forest plot showing pooled random‐effects estimates of the correlation between CR‐SJT scores and outcomes with higher construct congruence with the CR‐SJT. Observations are indicated by author, (year) [#], where the number indicates a unique outcome measure from the study. Each horizontal line represents an individual study effect size (Fisher's *z*) and 95% confidence intervals (CI); diamonds represent pooled estimates (random effects [RE] model). The overall (higher and lower construct congruence) pooled validity coefficient was *z* = 0.22 (95% CI, 0.16–0.28; *p* < 0.001). See eAppendix 2‐Codebook for Outcome Construct Congruence, and eTable 4‐Log for Outcome Construct Congruence for details. (b) Forest plot of effect sizes for outcomes with lower construct congruence. Forest plot showing pooled random‐effects estimates of the correlation between CR‐SJT scores and outcomes with lower construct congruence with the CR‐SJT. Observations are indicated by author, (year) [#], where the number indicates a unique outcome measure from the study. Each horizontal line represents an individual study effect size (Fisher's *z*) and 95% confidence intervals (CI); diamonds represent pooled estimates (random effects [RE] model). The overall (higher and lower construct congruence) pooled validity coefficient was *z* = 0.22 (95% CI, 0.16–0.28; *p* < 0.001). See eAppendix 2‐Codebook for Outcome Construct Congruence, and eTable 4‐Log for Outcome Construct Congruence for details.

**FIGURE 3:**
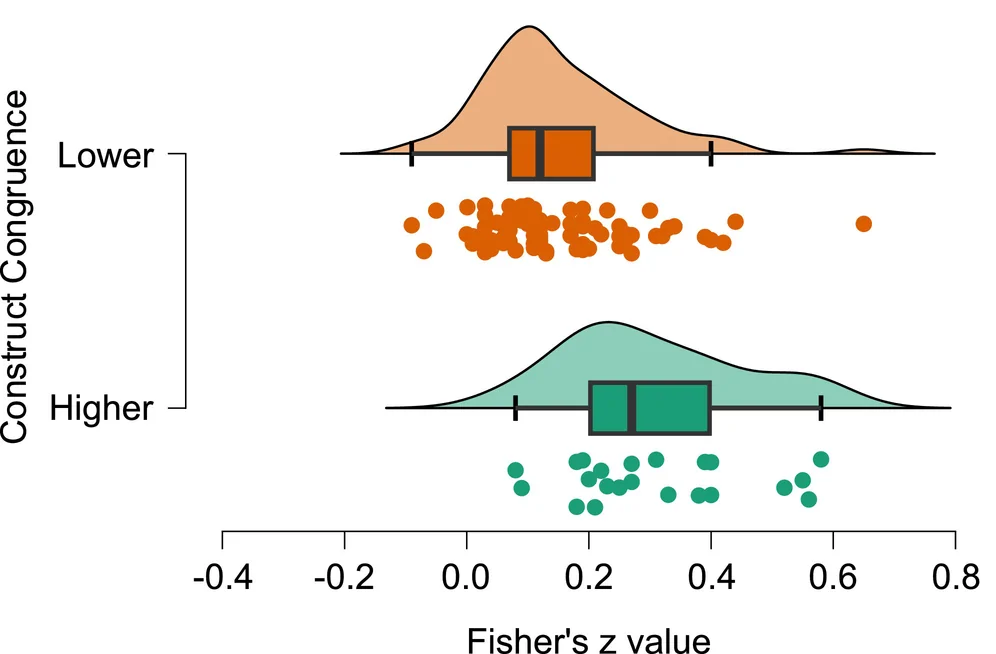
Raincloud plot of subgroup analysis by outcome construct congruence. Raincloud plot illustrating the distribution of individual observation correlations (Fisher's *z*) between CR‐SJT scores and outcome measures, stratified by outcome construct congruence (higher, lower). Each data point represents an effect size, with boxplots showing medians and interquartile ranges and half‐violins depicting density. The figure highlights consistently higher median correlations for higher‐congruence outcomes (*z* = 0.32, *n* = 22, 95% CI, 0.23–0.40; *p* < 0.001) compared with lower‐congruence outcomes (*z* = 0.17, *n* = 78, 95% CI, 0.11–0.24; *p* < 0.001), demonstrating construct‐related validity patterns. [Color figure can be viewed at wileyonlinelibrary.com]

**TABLE 1:** Results from univariate and multivariate meta‐regression analyses.

	Univariate (raw) coefficients		Multivariate (adjusted) coefficients	
Study factor	*B* (95% CI)	*p*‐value	*B* (95% CI)	*p*‐value
Higher construct congruence (versus lower)	0.13 (0.04, 0.22)	0.01*	0.13 (0.03, 0.23)	0.02*
Study type				
Grey literature (versus peer‐reviewed)	0.08 (−0.07, 0.23)	0.29	0.04 (−0.12, 0.21)	0.59
Grey literature (versus internal quality assurance)	0.08 (−0.07, 0.24)	0.25	0.07 (−0.08, 0.22)	0.32
Peer‐reviewed (versus internal quality assurance)	0.01 (−0.13, 0.14)	0.92	0.03 (−0.11, 0.16)	0.68
Outcome type				
Behavioural outcome (versus curriculum measure)	0.02 (−0.23, 0.27)	0.78	−0.04 (−0.22, 0.15)	0.58
Behavioural outcome (versus personality instrument)	0.07 (−0.18, 0.32)	0.40	0.04 (−0.14, 0.22)	0.55
Behavioural outcome (versus selection evaluation)	0.01 (−0.22, 0.25)	0.87	−0.03 (−0.24, 0.19	0.79
Behavioural outcome (versus work evaluation)	0.11 (−0.11, 0.33)	0.20	0.04 (−0.15, 0.23)	0.57
Curriculum measure (versus personality instrument)	0.05 (−0.30, 0.40)	0.40	0.08 (0.02, 0.13)	0.03*
Curriculum measure (versus selection evaluation)	−0.01 (−0.12, 0.10)	0.85	0.01 (−0.14, 0.16)	0.88
Curriculum measure (versus work evaluation)	0.09 (−0.08, 0.26)	0.10	0.07 (−0.16, 0.31)	0.18
Personality instrument (versus selection evaluation)	−0.06 (−0.43, 0.31)	0.37	−0.07 (−0.21, 0.08)	0.32
Personality instrument (versus work evaluation)	0.04 (−0.13, 0.20)	0.46	0.00 (−0.14, 0.14)	0.98
Selection evaluation (versus work evaluation)	0.10 (−0.10, 0.30)	0.15	0.07 (−0.14, 0.27)	0.44
Applicant (versus trainee) outcomes	0.02 (−0.09, 0.13)	0.66	−0.01 (−0.16, 0.14)	0.88

*Note*: Sample sizes for each study factor subgroup are as follows: Higher construct congruence (*N =* 22), lower construct congruence (*N* = 78), Grey literature (*N* = 32), peer‐reviewed (*N* = 44), internal quality assurance (*N* = 24), behavioural outcomes (*N* = 7), curriculum measures (*N* = 28), personality instruments *(N* = 7), selection evaluation (*N* = 41), work evaluation (*N* = 17), applicant outcomes (*N* = 45) and trainee outcomes (*N* = 55).

### 3.3 Risk of bias within studies

Using QUIPS, 52% (14) studies were rated at a low overall RoB, 45% (12) as moderate and 3% (1) as high. Domain‐level classifications are itemized in Supporting Information eTable 3‐QUIPS Risk‐of‐Bias Assessments for Included Studies. The sensitivity analysis, which compared the pooled effect with all studies to the effect after excluding the 14/100 with small sample sizes (*N* < 100) or a high RoB, was not significant (*p* = 0.435). After the exclusion, the pooled effect size was *z* = 0.23 (95% CI, 0.16–0.29; *p* < 0.001). A common source of bias was study confounding; many studies did not control for key confounders such as prior academic performance or sociodemographic variables. Furthermore, in some studies, the outcome measures were themselves confounded; combining academic knowledge with the interpersonal or professional skills, the CR‐SJT was intended to predict.

### 3.4 Publication bias

Visual inspection of the funnel plot (Figure 4) suggested symmetry, with no evidence of small‐study effects. Trim‐and‐Fill analyses using the R_0_, L_0_, and Q_0_ estimators revealed minimal evidence of publication bias with 0 missing estimates identified when L_0_ and Q_0_ estimators were implemented. The R_0_ estimator identified 6 missing estimates on the left‐hand side, adjusting the correlation from 0.22 to 0.16 (95% CI, 0.13–0.20). The overall effect remained significant, and the adjusted CI overlapped with the original estimate. Egger's regression‐based test confirmed the absence of significant publication bias (*t*(98) = 1.26, *p* = 0.211). Following the recommendations of Kepes et al. (2023) to avoid overreliance on any single method, the convergence of five out of six analyses (i.e., the publication type meta‐regression, the funnel plot, L_0_ and Q_0_‐based trim‐and‐fill analyses and Egger's regression‐based test) suggests that the results of this meta‐analysis were not significantly affected by publication bias.

**FIGURE 4:**
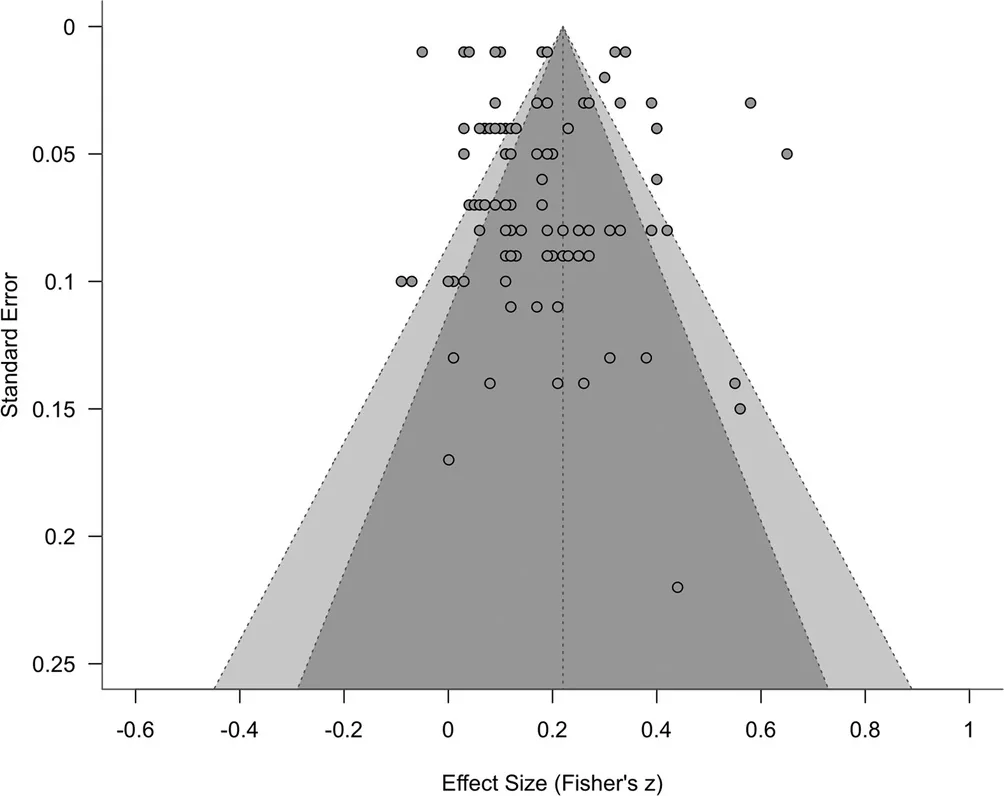
Funnel plot for assessment of publication bias. Funnel plot of standard error by fisher‐*z*‐transformed correlation coefficients assessing publication bias among included studies. The distribution is symmetrical around the pooled mean correlation *z* = 0.22 (95% CI, 0.16–0.28; *p* < 0.001), indicating minimal small‐study bias. Dashed vertical line indicates the pooled effect estimate; diagonal lines show pseudo‐95% confidence limits. Egger regression test was non‐significant (*t*(98) = 1.26, *p* = 0.211), supporting the absence of substantial publication bias.

### 3.5 Excluded studies

The meta‐analysis required studies to report a quantitative relationship between CR‐SJT scores and a downstream outcome measure. A number of identified studies did not meet this criterion due to limitations in data reporting or study design and were therefore excluded from the synthesis. However, given that the intended audience of this review includes health professions admissions officers and program administrators, these studies are identified here, as they reflect alternative evaluation designs that may be informative for programs evaluating operational validity beyond effect size magnitudes. Hosseinpour et al.^26^ reported comparisons to another applicant group rather than direct performance correlations. Another evaluated the frequency and severity of intervention for residents in difficulty pre and post CR‐SJT adoption.^27^ For Hadad et al,^28^ CR‐SJT performance was included as one of several metrics in combined assessment results without an ability to distinguish its contribution. One technical manual was identified and included a number of case studies reporting associations between CR‐SJT scores and various outcome measures.^11^ Programs were contacted directly related to these case studies. Only results retrieved through programme‐submitted quality assurance studies or other publications such as conference presentations were included in the meta‐analysis, leaving five technical manual case studies not included in this report.

## 4 DISCUSSION

This meta‐analysis provides the first quantitative synthesis of CR‐SJT validity evidence in health professions education. Across 27 studies and 100 effect sizes, CR‐SJT scores demonstrated a consistent, statistically significant association with personal and professional attributes. The strength of this association varied substantially by construct congruence: Effects were twice as large among outcomes with higher construct congruence (*z* = 0.32) compared to those with lower congruence (*z* = 0.17). The following sections interpret these findings relative to existing evidence, discuss their practical significance and outline limitations and priorities for future research.

### 4.1 Interpretation in context

A pooled correlation of 0.22 (95% CI, 0.16–0.28) was observed in this meta‐analysis. Webster et al reported a coefficient of 0.32 (95% CI, 0.26–0.39).^9^ Both estimates are total pooled values from three‐level random‐effects models, and in both analyses, substantial heterogeneity was observed in both meta‐analyses (Webster et al.: *I* ^2^ = 96.5%; present study: *I* ^2^ = 96%).^9^ Pooled estimates should be interpreted cautiously, considering the differences in study design, outcome selection and population across studies. These findings highlight the importance of determining relationships within specific contexts as opposed to assuming a ubiquitous effect. Nonetheless, this difference warrants discussion.

Webster et al. noted that postgraduate setting independently predicted larger validity coefficients (*β* = 0.23) in multivariable analysis. Nine of the ten largest validity coefficients in Webster et al. (*r* ≥ 0.50) were drawn from postgraduate specialty training, four of which were high‐fidelity simulation selection centre outcomes.^9^ These outcomes are contextually similar to the SJT, high‐stakes evaluation with higher construct congruence and often administered temporally proximal to one another. In contrast, this meta‐analysis drew almost exclusively from the undergraduate context, and only one of the one hundred effects was related to postgraduate selection. Instead, the present study included a larger proportion of selection outcomes, behavioural indicators in training (e.g., awards and leadership recognition) and personality instruments. Approximately 30% of effect sizes in the present analysis came from outcome categories not represented in Webster et al, and none were high‐fidelity simulation selection centre outcomes. These differences in outcome and sample composition are plausible explanations for the differences in pooled estimates. Beyond differences in sample and outcome composition, moderator analyses offered a more direct account of what drives validity in this context.

### 4.2 On the application of construct congruence

Trainee assessments in clinical contexts are typically multidimensional. They are shaped by cognitive ability, technical or clinical skill and contextual factors. In addition, the functional range of scores for both predictor and outcome can be limited, either by the nature of selection or by common practice in the program.^29^ Within this landscape, interpersonal and professional skills, even when they are explicit program priorities, remain difficult to specifically evaluate.

The CR‐SJTs predominantly evaluated in this meta‐analysis assess a specific capacity within the broader domain of personal and professional attributes: the ability to reason through and communicate a response to situations involving social and ethical uncertainty, in addition to the judgement or decision itself. The moderator analysis is consistent with this. Construct congruence between the CR‐SJT and the outcome was the only significant moderator of effect size, and the direction is theoretically expected.^10^, ^30^ Higher congruence outcomes yielded a pooled correlation of 0.32, compared with 0.17 for lower‐congruence outcomes. The more notable finding, however, is the disparity in representation. The majority of effects in this study fell into the lower category (78 compared to 22), reflecting that most evaluations do not isolate the interpersonal and professional skills that programmes presumably consider important. Webster et al. identified the development of construct‐explicit outcome measures as a pressing research need, and the present findings reinforce that recommendation.

For programs with a specific interest in selecting for or developing the capacity to reason through and communicate under social and ethical uncertainty, deliberate and curriculum‐embedded evaluation may be required. Targeted components of workplace‐based assessments, rather than global performance ratings, represent one feasible approach. This kind of intentional alignment between selection and training evaluation would support more meaningful programmatic decision‐making.

### 4.3 Practical implications

It is important to distinguish between concurrent and predictive evidence. Most (91%) of the outcomes followed a predictive design. This suggests that CR‐SJTs are related to performance in subsequent evaluations either in selection or training, fulfilling the immediate operational needs of the admissions office. Furthermore, when evaluating observed effect sizes, domain norms are critical.^31^ A meta‐analysis of over 140 000 effect size estimates reported a median observed correlation of *r* = 0.16 and a 75th percentile of *r* = 0.32, highlighting that correlations greater than ≈ 0.30 are uncommon in this domain.^32^ Similarly, Funder and Ozer recommend grounding effect size interpretation in both typically observed results and in the real‐world consequences. The authors therefore offered context‐based categories where *r* = 0.10 is small, 0.20 medium and 0.30 large.^33^ Regardless of the convention used to label an effect, the practical significance of that effect depends on its concrete consequences in decision‐making.

To illustrate these consequences, we apply the expectancy model to data reported in one of the included studies, using the TaylorRussell R package.^34^ The authors examined the association between CR‐SJT scores and competency‐based fieldwork evaluation (CBFE) performance for 120 trainees and found a correlation coefficient *r*_*s*_ = 0.279 for a single, second‐year placement assessment. The authors reported that the programme received 548 applications and made 179 offers (selection ratio = 0.327).^35^ The expectancy model therefore estimates that using the CR‐SJT in selection raises the proportion of above‐average CBFE performers from 50% to 62.4%. This would correspond to 22 additional above‐average CBFE performers among the 179 students offered admission. Substituting the pooled meta‐analytic estimate from the present study (0.22) yields a success ratio of 59.8%. In practical terms, this represents replacing 18 to 22 applicants who were likely to perform below average with applicants more likely to excel, translating to fewer students at risk of marginal fieldwork performance and fewer cases requiring faculty intervention. In programmes where support resources are limited, a 10%–12% improvement in cohort composition may carry meaningful administrative value.

Programs are encouraged to conduct utility analyses specific to their data and context. The practical significance of a validity coefficient varies substantially by institutional resources and constraints even if the value itself is unchanged. Analytic approaches including expectancy charts,^36^ Bayesian probability of superiority^37^ and systems‐based return on investment frameworks^38^ can provide the specificity needed to translate any observed effect size into an informed, context‐sensitive admissions decision.

### 4.4 Limitations

A primary limitation of this meta‐analysis is the potential impact of attenuation on the pooled estimate. Range restriction is a structural feature of admissions validity research, and all 100 effects are raw, unadjusted correlations. The degree to which correction would change this kind of estimate is actively debated. Sackett et al.^39^ suggest modest adjustment, conservatively 0%–30%, depending on the degree of range restriction and criterion unreliability. Critics of this view argue more substantial corrections are appropriate, at the lower bound ranging from 30% to over 70% under typical admissions conditions.^40^, ^41^ The raw association in the present study may be relatively accurate for outcomes with reliable criterion measures and broader sample representation, such as some selection evaluations, but substantially attenuated for highly selective contexts with unreliable workplace outcomes. Beyond magnitude, Sackett et al.^42^ emphasize that practical factors including candidate experience, cost and diversity implications should inform implementation decisions alongside validity estimates. To illustrate the practical consequences of attenuation, we extend the Taylor–Russell example presented earlier: A 30% and 70% corrected estimate would increase the projected cohort composition improvement from 18 to 23 and 30 candidates per 179 offered, respectively.

Substantial heterogeneity was observed across included studies (*I* ^2^ = 96%), reflecting variation in study designs, scoring methods, and outcome measures, and is consistent with heterogeneity reported in prior SJT meta‐analyses in this domain.^9^ This limits the precision of the pooled estimate and suggests results may not generalise uniformly across programmes or contexts. Further, two outcome type moderator categories should be interpreted with caution due to small samples (behavioural outcomes (*N* = 7) and personality instruments (*N* = 7)). Nearly half of studies had moderate (45%) or high (3%) risk of bias, particularly in participant selection and confounding domains, potentially introducing systematic error. However, sensitivity analyses excluding high‐risk studies did not materially alter the pooled estimate. Most included studies reported simple bivariate correlations without adjustment for confounders or examination of mediators, limiting conclusions about causal or incremental validity and differential attainment.^43^, ^44^ A formal certainty of evidence evaluation was not completed. Consistent with the heterogeneity and risk of bias findings noted above, confidence in the pooled estimate should be interpreted cautiously. That said, effects were consistently positive across studies, confidence intervals typically excluded zero, and no clear evidence of publication bias was identified across the five methods employed, providing some basis for confidence in the direction and approximate magnitude of the association.

Finally, despite efforts to capture the breadth of available evidence, grey literature and quality assurance reports, this synthesis is incomplete. As indicated in Section 3, some eligible studies could not be included because authors did not respond to data requests or provided insufficient information for effect size extraction. While these gaps are common to reviews of this kind, they constrain the conclusions that can be drawn.

### 4.5 Future directions

Of greatest consequence, the outcomes examined in this meta‐analysis are predominantly assessed during selection or training as opposed to post‐training patient‐related outcomes. The scarcity of professional role outcomes in our findings highlights a critical need for longitudinal infrastructure. As noted by Webster et al., national‐level databases linking applicant scores to long‐term professional outcomes would be essential for answering this question.^9^ While proximal outcomes in selection and training are informative for admissions office operations, it is still necessary to understand these tools related to longitudinal professional outcomes.

Next, advances in GenAI raise both challenges and opportunities. Several LLMs have matched or outperformed humans on SJTs assessing social and professional judgement,^45^ and ChatGPT has demonstrated high accuracy on knowledge‐based SJT formats assessing ethical competencies.^46^, ^47^ When unsupervised, these capabilities represent a meaningful threat to construct validity, though evidence suggests that assessment administration features such as time constraints, proctoring and operational restrictions may limit AI‐assisted performance.^48^ At the same time, LLMs offer genuine promise for automating item development, administering multimedia formats and scoring open‐ended responses at scale, developments that could address longstanding feasibility barriers to CR‐SJT adoption.^8^ As these technologies evolve, future validity research will need to account for AI both as a potential confounder and as an active design consideration in how CR‐SJTs are developed, delivered, and scored.

Finally, future primary studies would improve their operational validity and utility by more consistently reporting potential confounders such as sex and ethnicity and adjusting for proxies of cognitive ability. Prospective collection and reporting of demographic and contextual variables, combined with multivariable approaches such as regularized regression, would enable more rigorous evaluation of validity and differential attainment. This would facilitate more rigorous inference, similar to that demonstrated by Sahota et al.^49^

### 4.6 Conclusions

The use of CR‐SJTs in health professions selection is supported by available evidence. Validity is substantially conditioned on construct congruence between the CR‐SJT and the outcome measure, and most existing training evaluations do not isolate their evaluations for interpersonal and professional skills. Future research should prioritize developing construct‐explicit outcome measures and the longitudinal infrastructure needed to evaluate the professional impact of these tools.

## AUTHOR CONTRIBUTIONS

**Alexander MacIntosh:** Conceptualization; supervision; methodology; data curation; writing—original draft; writing—review and editing; project administration; formal analysis; investigation; validation. **Colin Henning:** Methodology; validation; visualization; writing—review and editing; writing—original draft; investigation; formal analysis; software; data curation. **Safea Altef:** Data curation; investigation; formal analysis. **Ivy Erickson:** Writing—original draft; writing—review and editing; validation. **Sobia Shariff Hussaini:** Writing—original draft; writing—review and editing; validation. **Elizabeth Morgan:** Writing—original draft; supervision; investigation; validation; writing—review and editing. **Yoon Soo Park:** Conceptualization; methodology; supervision; writing—review and editing. **Amir H. Sam:** Conceptualization; methodology; supervision; writing—review and editing.

## CONFLICT OF INTEREST STATEMENT

AM is a salaried employee and Scientist at Acuity Insights Inc., which develops a constructed‐response situational judgement test (CR‐SJT) used in health professions admissions. Their compensation is not related to the results of this study, and he holds no equity or performance‐based incentives from the company. All other authors report no conflicts of interest relevant to this work. No specific funding was received for this study. The work was undertaken as part of AM's employment at Acuity Insights Inc. Acuity Insights did not provide dedicated research funding for this project, and no external financial support was obtained.

## ETHICS STATEMENT

Ethical approval was not required for this study as it is a meta‐analysis of previously published and de‐identified data and did not involve direct interaction with human subjects

## Supporting information


eTables

eAppedices 

## Data Availability

The data that support the findings of this study are available in the Supporting Information of this article.
