# Supplementary material for: Validity of constructed‐response situational judgement tests in health professions education: A systematic review and meta‐analysis
Source: Med Educ. 2026 Jun 4;60(12):1319–31. doi: 10.1111/medu.70245 (PMC13736143; doi:10.1111/medu.70245)
Supplement: Supplementary file 1 [file MEDU-60-1319-s002.docx]

# Supplemental Material Contents

1. eAppendix 1 - Citations of Included Studies
2. eAppendix 2 - Codebook for Outcome Construct Congruence
3. eAppendix 3 - Outcome Type Descriptions and Examples

## eAppendix 1 - Citations of Included Studies

1.	Shipper ES, Mazer LM, Merrell SB, Lin DT, Lau JN, Melcher ML. Pilot evaluation of the Computer-Based Assessment for Sampling Personal Characteristics test. *J Surg Res*. 2017;215:211-218. doi:10.1016/j.jss.2017.03.054

2.	Woodham MB. *Examining the Validity of CASPer as a Medical School Admission Prescreening Assessment*. Trevecca Nazarene University; 2022. Accessed April 7, 2024. https://www.proquest.com/openview/8c3a4eb32679d40326fee7e1d63de786/1?pq-origsite=gscholar&cbl=18750&diss=y

3.	Dore KL, Reiter HI, Kreuger S, Norman GR. CASPer, an online pre-interview screen for personal/professional characteristics: prediction of national licensure scores. *Adv Health Sci Educ*. 2017;22(2):327-336. doi:10.1007/s10459-016-9739-9

4.	Dore KL, Reiter HI, Eva KW, et al. Extending the Interview to All Medical School Candidates—Computer-Based Multiple Sample Evaluation of Noncognitive Skills (CMSENS): *Acad Med*. 2009;84(Supplement):S9-S12. doi:10.1097/ACM.0b013e3181b3705a

5.	MacIntosh A, Mangardich H, Pan X, Goodell K. Incorporating a Constructed-Response Situational Judgment Test in a Medical School’s Holistic Review. In: *Proceedings of the 2023 AERA Annual Meeting*. AERA; 2023. doi:10.3102/2012320

6.	US-Med-A. *Program Quality Assurance Report*.; 2020.

7.	US-Med-B. *Program Quality Assurance Report*.; 2021.

8.	US-Med-C. *Program Quality Assurance Report*.; 2021.

9.	Roduta Roberts M, Alves CB, Chen F, St. Jean CR. Associations between an open-response situational judgment test and performance on OSCEs and fieldwork: implications for admissions decisions and matriculant diversity in an occupational therapy program. *BMC Med Educ*. 2024;24(1):1146. doi:10.1186/s12909-024-06071-0

10.	US-Med-D. *Program Quality Assurance Report*. East Tennessee State University; 2020.

11.	Knorr M, Mielke I, Amelung D, et al. Measuring personal characteristics in applicants to German medical schools: Piloting an online Situational Judgement Test with an open-ended response format. *GMS J Med Educ*. 2024;41(3). doi:10.3205/ZMA001685

12.	Lehman D, Mangardich H, Sisco B. An Analysis of a DPT Program’s Use of Casper as an Admissions Decision Criteria. Poster presented at: APTA Combined Sections Meeting (CSM); 2024; Boston, MA. Accessed April 7, 2024. https://apta.confex.com/apta/csm2024/meetingapp.cgi/Paper/41823

13.	Robb C, Copeland L, Banks P, MacIntosh A, Dore K. Predictive Validity of an SJT, Casper, with Both Typed and Video Responses. Presented at: Ottawa; 2024; Melbourne, Australia. Accessed April 7, 2025. https://qmec.org/cms/Microsite/content.html

14.	Juster F, Petersen KH, Dore KL. Holistic Admissions to Holistic Success: How do Admissions Tools Predict Broad, Holistic Outcomes of Success in UME? Poster presented at: AAMC LSL; November 3, 2023; Seattle, WA. Accessed April 7, 2024. https://web.cvent.com/event/54416737-ee96-45ef-9f04-6b23efb58591/summary

15.	US-Vmed. *Program Quality Assurance Report*. Google Docs; 2023.

16.	Davis M, Smith-Trigg E. Admitting the Successful Student: SJT’s and PreclinicalProfessionalism Measures. Presented at: AACOM Educating Leaders; April 24, 2023; Baltimore, MD. Accessed April 7, 2025. https://www.aacom.org/docs/default-source/events/2023-el/el23-conference-program-book.pdf?sfvrsn=426b95d3_1

17.	Proscia-Lieto T, Fanning SL. Correlation of Casper with humanistic outcomes in an osteopathic medical program. Presented at: Admissions Summit; June 7, 2021; Virtual.

18.	Woodson M, Hussin A, MacIntosh A. Evaluating Medical School Admissions Screening Measures to Promote Diversity While Relating to Future Clerkship Performance. In: *Proceedings of the 2022 AERA Annual Meeting*. AERA; 2022. doi:10.3102/1887714

19.	Van Rens SM, Henning CT, Crane AG, MacIntosh A, Sitarenios G, Parker JDA. Validating a short measure of personal intelligence: A multimethod assessment approach. *Personal Individ Differ*. 2025;237:113052. doi:10.1016/j.paid.2025.113052

20.	Yingling S, Park YS, Curry RH, Monson V, Girotti J. Beyond cognitive measures: Empirical evidence supporting holistic medical school admissions practices and professional identity formation. *MedEdPublish*. 2018;7:274. doi:10.15694/mep.2018.0000274.1

21.	Parker-Newlyn L, Mansfield K, Dore K. CASPer Down Under: understanding the role of SJT in selecting rural background applicants for medical school in Australia. *Can Med Educ J*. 2020;11:e77. doi:https://doi.org/10.36834/cmej.v11i2

22.	Yan XW, Wood T, Lemay G. Is the Casper test a good predictor of medical student outcomes? *Can Med Educ J*. 2021;12:e72. doi:https://doi.org/10.36834/cmej.72402

23.	Yan XW, Lemay G, Wood T. Does Casper predict medical school performance? *CCME 2022 Conf*. Published online 2022. doi:https://doi.org/10.36834/cmej.75002

24.	Rothnie I, Dore K, Derby J, Starenios G, Kostrz K, Newton L. Validity evidence for a Situational Judgement Test as a measure of professionalism in selection processes for a post graduate vocational medical training program. Presented at: Ottawa; 2024; Melbourne, Australia. Accessed April 7, 2025. https://qmec.org/cms/Microsite/content.html

25.	Reiter HI, Zou C, Juster F, Dore K. The Validity of a Situational Judgment Test for Medical School Admissions. Presented at: SIOP; 2019; Washington, DC. Accessed April 7, 2024. https://www.siop.org/wp-content/uploads/legacy/Conference/2019/programFINAL.pdf?ver=2019-03-07-132243-557

26.	US-Med-E. *Program Quality Assurance Report*. University of Vermont; 2025. https://streaming.uvm.edu/private/videos/9G6quaW/

27.	Maher S, Parnell R, Moore EW, Frade P, Markman B. Computer-Based Assessment of Non-Cognitive Attributes of Occupational Therapy Students: A Pilot Evaluation. *Internet J Allied Health Sci Pract*. Published online January 4, 2023. doi:10.46743/1540-580X/2023.2249

## eAppendix 2 - Codebook for Outcome Construct Congruence

A structured coding scheme was used to assign a level of construct congruence between the CR-SJT and the outcome measure. Outcome measures were assigned as *higher* in construct congruence when greater than half the measure was described as overlapping with characteristics assessed by the CR-SJT. Conversely, outcome measures were assigned as *lower* in construct congruence when less than half the measures score was derived from aspects associated with the CR-SJT.

The following coding rules were applied to assign alignment status to each outcome measure. The **S6 - eTable 4 - Log for Outcome Construct Congruence** lists each outcome measure and its extracted rational or reference from the article.

**To Classify Each Outcome:**

1. Review and extract each outcome measure’s description in the article or its referenced source.
2. Identify aspects of the outcome measure that do and do not conceptually overlap with the CR-SJT.
3. Determined whether the outcome score meaningfully overlapped with the domains measured by the SJT.

**Criteria for *Higher* Construct Congruence**

- The outcome measure assesses *multiple or most* of the interpersonal or professional competencies like the CR-SJT used. That is, a substantial portion of the measure’s score is derived from overlapping competencies.
- Examples:
  - Total Multiple Mini-Interview (MMI) score with stations evaluating communication, collaboration, resilience.
  - The self-reported assessment: Multidimensional Inventory of Personal Intelligence (MIPI) assessing 3 domains: social, motivational, and emotional intelligence.
  - The subsection of the Medical Council of Canada Qualifying Examination (MCCQE-I) evaluating: Communication, legal, ethical organizational (CLEO).
- Note – Include outcomes where the article specifically articulates significant or meaningful overlap in construct congruence between the CR-SJT and the outcome measure.

**Criteria for *Lower* Construct Congruence**

- The outcome measure assesses *one or few* of the interpersonal or professional competencies like the CR-SJT used. That is, a small portion of the measure’s score is derived from overlapping competencies.
- Examples:
  - Applicants rank list position incorporating GPA, MCAT
  - Overall Clerkship performance rating considering clinical skill and professionalism
  - Defining Issues Test (a measure of moral reasoning, DIT2), a measure of moral judgment, one central component of moral behavior
  - MCAT- Critical Analysis and Reasoning Skills (CARS) which asks the exam-taker to critically analyze information provided in passages. Includes content from ethics, philosophy, studies of diverse cultures.
- Note – This will usually be outcomes with predominantly academic or technical focus. However, if a subcomponent (e.g., a single communication sub-score in an Objective Structured Clinical Examination (OSCE)) exists, but the overall SJT score emphasizes a much larger group of components (e.g. communication teamwork, resilience, ethics, collaboration…), it should be marked low, since only one part of the CR-SJT is represented in the outcome (e.g. communication).

**Note**

Classification of congruence between CR-SJT constructs and outcome measures was inherently interpretive. Although coding followed a predefined framework, most studies provided limited detail regarding construct definitions or scoring rationales. This required subjective judgment, particularly in cases of partial overlap or inconsistent labeling across instruments. These groupings were not derived from statistical analyses (e.g., factor analysis or structural equation modeling) but reflect qualitative synthesis. All disagreements were resolved through discussion among coders.

## eAppendix 3 - Outcome Type Descriptions and Examples

| **Outcome Type** | **Description of Commonality** | **Items Included** |
| --- | --- | --- |
| **Selection Evaluations** | Outcomes determined during the admissions process to evaluate the suitability of applicants for entry into health professions programs. | Multiple Mini-Interview (MMI) total and sub-scores (e.g., compassion, morality, cultural, sociability, self-awareness); faculty interviews; admissions offers (offer, rejection, withdrawal); applicant rank list position; invitation to interview; Admissions portfolio score; Hamburger Situational Judgement Test (HAM-SJT); Critical Analysis and Reasoning Skills (CARS) component of MCAT. |
| **Curricular Assessments** | Standardized performance measures within the academic program, including course grades, clinical skills examinations, and national licensure exams. | Objective Structured Clinical Examination (OSCE) total and station scores; Course grades (Clinical Skills, Medicine, Psychiatry); Medical Council of Canada Qualifying Examination (MCCQE) sub-scores (CLEO, PHLEO, Patient Interaction, Total); Clerkship Clinical Competency Evaluation (CCCE) scores; Peer-Evaluations; Professionalism ratings; Professional Identity Essay (PIE). |
| **Workplace Evaluations** | Formal evaluations of performance conducted in clinical or workplace settings, typically through supervisor ratings or competency-based forms. | Clerkship grades (e.g., Honors, High Pass); Competency-based fieldwork evaluation items (e.g., professional interactions, communication, professional development, performance management). |
| **Behavioural Outcomes** | Discrete observations of conduct, often binary or count-based indicators related to professionalism domains such as leadership, awards, remediation that occur outside of standardized rating scales. | Documented professionalism lapses or handbook violations; assumption of leadership roles; documented community service hours; receipt of clinical or service excellence awards. |
| **Personality Instruments** | Standardized psychological measures or surveys assessing stable personal traits, values, or moral reasoning styles. | Multidimensional Inventory of Personal Intelligence (MIPI) subscales (Emotional, Social, Motivational, and Personal Intelligence); Defining Issues Test (DIT-2) indices (Personal Interest, Maintain Norms, Post-Conventional). |
